# Diet and physical exercise as key players to tackle MASLD through improvement of insulin resistance and metabolic flexibility

**DOI:** 10.3389/fnut.2024.1426551

**Published:** 2024-08-20

**Authors:** Sara Paola Mambrini, Antonio Grillo, Santo Colosimo, Francesco Zarpellon, Giorgia Pozzi, Davide Furlan, Gabriele Amodeo, Simona Bertoli

**Affiliations:** ^1^Nutrition Science Research Lab, Ospedale S. Giuseppe, Istituto Auxologico Italiano IRCCS, Piancavallo, Italy; ^2^Istituto Auxologico Italiano IRCCS, Piancavallo, Italy; ^3^Department of Food, Environmental and Nutritional Sciences (DeFENS), University of Milan, Milan, Italy; ^4^PhD School of Nutrition Science, University of Milan, Milan, Italy

**Keywords:** MASLD, physical exercise, insulin resistance, metabolic flexibility, Mediterranean diet, resistance exercise

## Abstract

Metabolic Dysfunction-Associated Steatotic Liver Disease (MASLD) has emerged as a prevalent health concern, encompassing a wide spectrum of liver-related disorders. Insulin resistance, a key pathophysiological feature of MASLD, can be effectively ameliorated through dietary interventions. The Mediterranean diet, rich in whole grains, fruits, vegetables, legumes, and healthy fats, has shown promising results in improving insulin sensitivity. Several components of the Mediterranean diet, such as monounsaturated fats and polyphenols, exert anti-inflammatory and antioxidant effects, thereby reducing hepatic steatosis and inflammation. Furthermore, this dietary pattern has been associated with a higher likelihood of achieving MASLD remission. In addition to dietary modifications, physical exercise, particularly resistance exercise, plays a crucial role in enhancing metabolic flexibility. Resistance exercise training promotes the utilization of fatty acids as an energy source. It enhances muscle glucose uptake and glycogen storage, thus reducing the burden on the liver to uptake excess blood glucose. Furthermore, resistance exercise stimulates muscle protein synthesis, contributing to an improved muscle-to-fat ratio and overall metabolic health. When implemented synergistically, the Mediterranean diet and resistance exercise can elicit complementary effects in combating MASLD. Combined interventions have demonstrated additive benefits, including greater improvements in insulin resistance, increased metabolic flexibility, and enhanced potential for MASLD remission. This underscores the importance of adopting a multifaceted approach encompassing dietary modifications and regular physical exercise to effectively manage MASLD. This narrative review explores the biological mechanisms of diet and physical exercise in addressing MASLD by targeting insulin resistance and decreased metabolic flexibility.

## 1 The high prevalence of MASLD urges effective treatment options based on optimization of lifestyle changes

Metabolic Dysfunction-Associated Steatotic Liver Disease (MASLD) is a prevalent condition that affects approximately 25% of the global population (1). It is commonly considered as the liver-related manifestation of the metabolic syndrome and is closely linked to obesity and type 2 diabetes (T2D) (2). MASLD is a disease spectrum spanning from simple excess triacylglycerol (TAG) accumulation within the hepatocytes (steatosis) to inflammation (metabolic dysfunction-associated steatohepatitis, MASH), cirrhosis and hepatocellular carcinoma (HCC) (3). MASLD carries a burden of morbidity and mortality, primarily due to liver-related complications, but more significantly due to unfavorable cardiovascular events. Glucose lowering drugs based on the incretin system are often used off-label to promote the optimization of glucose control when type 2 diabetes is an issue, or for the management of weight loss (4, 5). The primary approach to managing the disease involves substantial weight loss and intensive reduction of cardiovascular risk factors.

Nonetheless, lifestyle changes aimed at weight loss remain the mainstay of MASLD treatment in general population.

As far as weight loss for the treatment of MASLD is concerned, the recommendation is a targeted weight reduction of 7–10%, a measure found to be effective in mitigating lipid accumulation, enhancing metabolic flexibility, and ameliorating insulin resistance (6).

Weight loss exceeding 10% has demonstrated superior efficacy in resolving MASLD and MASH, exhibiting regression of fibrosis and inflammation (6, 7). Notably, the synergistic benefits of weight loss are contingent on the adoption of a combined dietary and exercise approach (8).

Resmetirom, an oral, liver-directed, thyroid hormone receptor beta (THR-β)–selective agonist, were superior to placebo with respect to MASH resolution and improvement in liver fibrosis in a stage 3 trial (9). Resmetirom may represent the first approved liver specific drug aimed at MASH resolution (10), on top of lifestyle changes aimed at weight loss.

With this article we would like to review the mechanisms by which dieting and physical exercise may benefit metabolic health and fatty liver through improved insulin resistance and metabolic flexibility. While insulin resistance has been considered one of the most relevant pathogenetic feature of MASLD and the driver for the progression of liver inflammation (11), reduced metabolic flexibility remains a less explored mechanism (12).

### 1.1 Energy expenditure at rest and metabolic adaption as determinants of weight loss programs success

Basal metabolic rate (BMR) refers to the amount of energy expended by an organism during a state of physical and psychological rest, measured in the morning while fasting (after a 10-h period since the last meal), without preceding physical exertion after eating, and in a thermoneutral environment (13).

Understanding how human bodies oxidize calories at rest can help us understand why some people are more susceptible to MASLD. Conditions like obesity and diabetes, which are linked to BMR reduction, are also known to contribute to MASLD. However, the exact impact of BMR on MASLD needs further investigation (14, 15).

In individuals who lead a sedentary lifestyle, BMR accounts for a significant proportion, ranging from 60 to 70% of the total energy expenditure, whereas in those who regularly engage in physical activities, this percentage decreases to approximately 50% (16).

The factors that influence basal metabolism have demonstrated their crucial role, as changes in these parameters have been causatively associated with an increased susceptibility to specific clinical conditions, such as obesity, thereby raising the risk of developing MASLD (17).

According to existing literature, modifications in lean mass and adipose tissue mass are recognized as the primary influencers of BMR (13).

Contemporary research is increasingly focused on developing strategies for weight loss in individuals with overweight or obesity, while simultaneously preserving lean mass, which plays a significant role in basal energy expenditure. Conversely, divergent studies have revealed that after periods of calorie restriction, BMR may exhibit an adaptive response known as “metabolic adaptation,” which negatively affects the rate of BMR and predisposes individuals to a higher risk of weight regain (17–19).

The precise mechanisms underlying metabolic adaptation remain incompletely understood, although it is hypothesized to potentially involve reduced sympathetic drive or decreased thyroid activity (20). Caloric intake, dietary patterns and specific dietary components influence BMR. For instance, sufficient protein intake is considered crucial for maintaining lean mass in low-calorie dietary regimens (21, 22).

Additional factors that have emerged in the literature as influential in modulating BMR include exercise, height, general weight, age, and circulating thyroid hormones (23).

Physical activity has a profound impact on the rate of BMR by affecting changes in lean mass among individuals (see section 4). The influence of thyroid hormones on BMR has long been acknowledged, with hyperthyroidism or hypothyroidism conditions exerting noticeable effects on BMR. A recent study demonstrated that elevated plasma concentrations of T4 were associated with increased BMR. The role of triiodothyronine (T3) in this context remains a subject of debate (23).

### 1.2 Definition of metabolic flexibility

In order to understand the regulatory mechanisms of metabolism at rest, it is helpful to introduce the concept of metabolic flexibility. Metabolic flexibility refers to the body intrinsic ability to utilize readily available substrates as an energy source. During periods of rest, the body can shift between using glucose and lipids as primary fuel sources. After a meal, insulin promotes the storage of glucose as glycogen in the liver and facilitates glucose uptake in skeletal muscle. Conversely, during fasting conditions, glucagon stimulates hepatic gluconeogenesis and glycogenolysis, while also promoting lipolysis in adipose tissue to release free fatty acids for use as an energy substrate. This coordinated regulation by insulin and glucagon allows the body to maintain metabolic homeostasis by adapting its fuel utilization to the prevailing nutritional state.

Disruptions to this metabolic flexibility, such as in metabolic disorders like MASLD, can impair the body ability to efficiently switch between glucose and lipid oxidation. This can lead to the accumulation of lipids in non-adipose tissues like the liver, exacerbating disease progression.

### 1.3 Regulatory mechanisms of energetic metabolism at rest revolve around insulin and glucagon insulin

#### 1.3.1 Insulin

Metabolic regulation during periods of rest involves complex molecular signaling pathways.

We begin our examination with the role of insulin. After a meal, pancreatic β cells release insulin into the bloodstream, resulting in an elevated insulin-glucagon ratio. Insulin exerts its effects on various tissues, promoting glycolysis and glycogen synthesis in the liver while inhibiting gluconeogenesis and glycogenolysis. In skeletal muscle, insulin facilitates glucose uptake by translocating the insulin-dependent glucose transporter GLUT 4 to the plasma membrane, and it also promotes glycogen synthesis and glycolysis. Moreover, in adipose tissue, insulin, upon binding to its receptor, suppresses lipolysis while simultaneously stimulating the production of lipids (24).

Additionally, insulin exerts its effects by promoting protein synthesis, regulating mitochondrial biogenesis, and inhibiting autophagy (25). This hormone plays a crucial role in the regulation of glucose homeostasis and the storage of energy in the form of lipids and glycogen. Acting as a peptide hormone, insulin interacts with the tyrosine kinase receptor (INS-R), initiating a signaling cascade that encompasses the aforementioned effects.

#### 1.3.2 Glucagon

Glucagon is released by pancreatic α-cells and acts as antagonist to insulin. Glucagon secretion is stimulated during fasting conditions and primarily exerts its effects at the hepatic level by promoting gluconeogenesis, glycogenolysis, lipolysis, and ketogenesis. Simultaneously, glucagon inhibits the pathways activated by insulin, such as glycolysis and lipogenesis. The decrease in insulin levels during fasting conditions suppresses malonyl-coenzyme A synthesis in liver cells, leading to the activation of fatty acid oxidation as the predominant energy source (26).

The regulation of energy homeostasis in the body relies on the interplay between insulin and glucagon. The contribution of gut-secreted incretin hormones, such as GLP-1, in stimulating insulin secretion has been extensively demonstrated in mechanicistic and clinical studies (27–29). Furthermore, there is evidence suggesting a reciprocal relationship, where glucose-induced insulin secretion inhibits glucagon secretion from α cells in a paracrine manner (26).

#### 1.3.3 Mitochondria

Insulin inhibits enzymes responsible for fatty acid oxidation, thereby directing mitochondria to preferentially utilize glucose as a fuel source.

When glucagon levels rise during fasting or starvation, the hormone signals mitochondria to shift away from glucose oxidation and toward increased fatty acid beta-oxidation.

Disruptions in mitochondrial function can lead to diminished energy production and an increased tendency for the accumulation of lipids within the liver (30). Dysfunctional mitochondria exhibit reduced efficiency in oxidizing fatty acids, resulting in an overflow of lipids and the formation of toxic lipid intermediates such as diacylglycerols and ceramides. These lipid species interfere with insulin signaling pathways, compromising the ability of hepatocytes to respond appropriately to insulin and regulate glucose metabolism (31).

### 1.4 Insulin resistance and gut-liver axis

Insulin resistance is a pathophysiological condition characterized by the decreased ability of target tissues, such as the liver, skeletal muscle, and adipose tissue, to respond appropriately to insulin (10). This impairment in insulin signaling and action results in impaired glucose and lipid metabolism (11). Insulin resistance can manifest at both the systemic and hepatic levels. Systemic insulin resistance refers to a whole-body phenomenon, where peripheral tissues exhibit reduced sensitivity to insulin, leading to hyperglycemia and compensatory hyperinsulinemia (11). In contrast, hepatic insulin resistance is the specifically impaired insulin action within the liver, which fails to properly suppress gluconeogenesis and increase glucose uptake in response to insulin. Hepatic insulin resistance contributes to the dysregulation of glucose and lipid homeostasis, further exacerbating metabolic disturbances and the development of MASLD.

Concurrently, targeting the gut-liver axes can improve systemic insulin sensitivity. The gut microbiome plays a pivotal role in this gut-liver axis, influencing metabolic flexibility through several mechanisms (12). Gut bacteria produce a variety of metabolites, including short-chain fatty acids (SCFAs), that can modulate hepatic and peripheral insulin sensitivity. SCFAs, for instance, can activate AMP-activated protein kinase (AMPK) in the liver, enhancing mitochondrial fatty acid oxidation and glucose metabolism (13) (see section 1.5).

Furthermore, the gut microbiome shapes the production of bile acids, which act as signaling molecules to regulate energy homeostasis. Specific bile acid species can activate the Farnesoid X Receptor (FXR) and G-protein coupled bile acid receptor (TGR5) in the liver, leading to improved gluconeogenesis, lipid metabolism, and insulin sensitivity (14, 15). Dysbiosis of the gut microbiome, as seen in MASLD, can disrupt this delicate balance and contribute to impaired metabolic flexibility (16).

Targeting the gut-liver axis through dietary interventions, prebiotics, probiotics, or pharmacological modulation of the microbiome and bile acid signaling may therefore represent a promising strategy to improve metabolic flexibility and mitigate insulin resistance in MASLD patients.

### 1.5 PGC-1α is a sensor of energy expenditure

Peroxisome Proliferator-Activated Receptor Gamma Coactivator-1alpha (PGC-1α) is a critical regulator of cellular processes and energy metabolism. Its expression is highest in tissues that have a high capacity for oxidative metabolism, such as skeletal muscle, liver, brown adipocytes, and myocardium (32). The role of PGC-1α varies depending on the specific tissue. In the liver, extensive research has been conducted on the effects of PGC-1α on mitochondrial and energy metabolic activities of hepatocytes. During fasting, there is an increase in the ratio of AMP to ATP, which activates AMPK and subsequently leads to the activation of PGC-1α. The primary effect of PGC-1α activation is the induction of mitochondrial biogenesis through its stimulation of nuclear transcription factors NRF1 and NRF2 (33). These transcription factors control the expression of important genes involved in energy metabolism, leading to increased expression of mitochondrial proteins and enhanced enzymatic capacity in oxidative metabolic pathways such as beta-oxidation, the tricarboxylic acid cycle, and oxidative phosphorylation (34). Furthermore, PGC-1α activation also induces the expression of antioxidant molecules that protect against reactive oxygen species (ROS) (34).

In individuals with obesity and insulin resistance, the liver is chronically exposed to high levels of free fatty acids from the portal circulation. Additionally, hyperinsulinemia, which is associated with insulin resistance, promotes the synthesis of fatty acids in the liver. These conditions contribute to the excessive accumulation of fatty acids, surpassing the mitochondrial oxidative capacity of hepatocytes and resulting in MASLD. In the context of MASLD, appropriate levels of PGC-1α play a positive role. This is because an increase in both mitochondrial mass and function has been observed, leading to enhanced beta-oxidation. Studies have shown a correlation between reduced levels of PGC-1α and an increased risk of developing hepatic steatosis, which can be attributed to impaired interaction with NRF promoters (34). This impaired interaction leads to decreased levels of mitochondrial proteins and antioxidants, resulting in further increased oxidative stress (35). Both *in vivo* and *in vitro* studies have demonstrated that overexpression of PGC-1α leads to increased hepatic beta-oxidation, causing a significant reduction in hepatocyte triglyceride accumulation. Exercise has also been identified as a useful tool for increasing PGC-1α levels in the liver, as well as in skeletal muscle tissue (36).

In skeletal muscle, contraction plays a crucial role in shaping its characteristics, including mitochondrial content and function, gene transcription, and intracellular signaling related to contractile proteins (12). However, in individuals with obesity and insulin resistance or T2D, there is a limited ability to switch from glucose oxidation to fatty acid oxidation, impairing metabolic flexibility (12). This is influenced by factors such as increased free fatty acid (FFA) intake, which leads to elevated mitochondrial acetyl CoA and cytosolic citrate levels. The accumulation of citrate inhibits phosphofructokinase, resulting in increased glucose-6-phosphate and inhibition of glycolysis by suppressing glucokinase. Furthermore, metabolic by-products of FFA degradation, such as ceramides and diacylglycerol, interfere with insulin receptor signaling, hindering GLUT 4 translocation to the cell membrane and exacerbating insulin resistance (37).

Elevated plasma FFA levels and increased muscle lipid content contribute to insulin resistance by disrupting mitochondrial regulation and promoting ectopic fat deposition (12). In the skeletal muscle of people with obesity, there is a predominance of type IIB fibers characterized by lower mitochondrial density and oxidative capacity, along with reduced expression of PGC-1α, a key regulator of mitochondrial biogenesis. PGC-1α is strongly induced by muscle contraction and, through positive feedback, upregulates the expression of MEF2, promoting myocyte enhancement. People with insulin resistance and obesity generally exhibit decreased expression of PGC-1α and NRF-1 in myocytes, resulting in reduced oxidative phosphorylation capacity and impaired fatty acid oxidation (38). Specifically, individuals with type 2 diabetes and glucose intolerance exhibit inhibited PGC-1α transcription, leading to reduced mitochondrial protein expression (39).

Intervention studies have shown that physical activity can positively impact mitochondrial metabolism and improve metabolic flexibility. In one study, aerobic exercise in elderly prediabetic subjects led to enhanced metabolic flexibility, as evaluated by the respiratory quotient during a hyperinsulinemic euglycaemic clamp (40). Considering the various roles of PGC-1α in energy metabolism, its induction by physical activity, and its reduced expression in obesity, diabetes, and insulin-resistance, it is evident that PGC-1α plays a critical role in the metabolic flexibility of skeletal muscle tissue.

White adipose tissue (WAT) serves not only as an energy reservoir in the form of triglycerides but also as an active regulator of metabolic health and substrate flow. Excessive WAT accumulation and dysfunction are closely associated with metabolic impairments, contributing to adipose-related diseases (12). Similar to muscle tissue, adipose tissue relies on insulin for its proper functioning. Insulin binding to its receptor initiates a phosphorylation cascade that ultimately leads to the translocation of GLUT4-containing vesicles to the cell membrane, facilitating glucose uptake. Insulin also inhibits lipolysis, reducing the release of non-esterified fatty acids (NEFA) into the circulation (41). However, insulin-resistant individuals experience dysregulation in these tightly controlled mechanisms, resulting in impaired modulation of lipolysis and persistent release of NEFA into the bloodstream (12).

There are two main types of adipose tissue: white adipose tissue (WAT) and brown adipose tissue (BAT). Brown adipocytes, characterized by their smaller size, rich cytoplasm, and high mitochondrial density, exhibit a remarkable oxidative capacity. The high expression of uncoupling protein 1 (UCP1) in brown adipocytes enables thermogenesis by dissipating the proton gradient in the mitochondrial intermembrane space, generating heat instead of ATP (42).

Studies have shown that white adipocytes can undergo conversion to brown-like adipocytes, and the key transcriptional factors involved in this process are PPAR-γ and PGC-1α (43). Treatment with rosiglitazone, a PPAR-γ agonist, has been found to significantly increase the expression of UCP1 and PGC-1α (44). PGC-1α, a coactivator, plays a crucial role in thermogenesis during cold exposure by increasing the AMP/ATP ratio, which activates AMPK. AMPK then phosphorylates PGC-1α, orchestrating thermogenesis and oxidative metabolic processes. Analysis of subcutaneous WAT in individuals with obesity reveals downregulation of PGC-1α (45). Moreover, experiments in mice with a specific deletion of PGC-1α in adipose tissue demonstrate reduced gene expression related to oxidative phosphorylation, beta-oxidation, glucose tolerance, and insulin resistance (46).

It is worth noting that aerobic and resistance exercise promotes increased lipolysis in white adipose tissue, leading to reduced adiposity and altered expression of key proteins involved in energy metabolism, such as the GLUT4 transporter and PGC-1 coactivator (46).

### 1.6 PPARα as crucial regulator of energy metabolism gene expression

PCG-1α can activate Peroxisome proliferator–activated receptor alpha (PPARα) through a complex regulatory mechanism. PCG-1α interacts with PPARα and enhances its transcriptional activity by acting as a coactivator. PCG-1α binds to PPARα and promotes the recruitment of other coactivators, such as CBP/p300, leading to the activation of target genes.

The activation of PPARα by PCG-1α has important hepatic effects. PPARα plays a crucial role in regulating lipid metabolism in the liver. When activated by PCG-1alpha, PPARα promotes fatty acid oxidation, leading to increased mitochondrial beta-oxidation and subsequent energy production. This activation also stimulates the expression of genes involved in fatty acid transport and metabolism, as well as ketogenesis.

PPARα is a transcription factor belonging to the nuclear receptor family. It is known for its affinity for various endogenous ligands, primarily lipid molecules such as fatty acids, prostaglandins, leukotrienes, and fatty acid-derived metabolites, as well as antidiabetic drugs (47). PPARα is predominantly found in tissues with high metabolism and energy demand, such as the liver, heart, skeletal muscle, and kidney. However, its presence in other tissues, including adipose tissue, highlights its significant role in lipid metabolism regulation (48).

PPARα plays a central role in lipid metabolism by regulating a key molecular pathway involved in beta-oxidation. It stimulates the expression of enzymes such as CPT1 and acyl-CoA oxidase (ACOX), which are crucial for the transport and subsequent oxidation of fatty acids within the mitochondrial matrix (49). Activation of PPARα also leads to the transcription of genes responsible for ketogenesis and bile acid metabolism during fasting, promoting the synthesis of ketone bodies (50). Additionally, PPARα positively affects lipoprotein metabolism by regulating the expression of apolipoproteins A1 and A2 (APOA1, APOA2), leading to increased plasma levels of these proteins and contributing to the modulation of HDL (51).

PPARα is involved in the regulation of the inflammatory response by negatively regulating leukotriene synthesis and trans-repressing the activities of inflammatory regulators such as NF-kB, AP-1, and STAT. This overall reduces the expression of inflammatory molecular pathways (52). Interestingly, an inverse correlation is observed between PPARα and the development of NASH. This suggests that increased inflammation may contribute to the suppression of PPARα expression (53).

Dysregulation of PPARα could have important consequences in hepatic lipid accumulation and increase the risk of cardiovascular disease. Therefore, PPARα could be a therapeutic target for resolving altered lipid metabolism.

### 1.7 PPARα functions as a lipid sensor in white adipose tissue

PPARα serves as a critical lipid sensor that coordinates the transcriptional response to fatty acids and plays a vital role in controlling oxidative capacity and thermogenesis, especially in brown adipose tissue. As such, PPARα is also expressed in adipose tissue and regulates several metabolic processes, including lipolysis, beta-oxidation, adipocyte differentiation, thermogenesis, and the inflammatory response. Activation of PPARα induces the expression of molecules involved in lipid metabolism.

PPARα functions as a lipid sensor, as it increases the transcription of PPARα-regulated genes in response to elevated fatty acid levels and chronic stimulation of beta-adrenergic receptors. This leads to an increase in oxidative capacity and thermogenesis (54). PPARα is mainly expressed in brown adipose tissue and has low expression in white adipocytes. However, studies have shown a negative correlation between PPARα mRNA levels in human adipose tissue and body mass index (55). Interestingly, some studies have found that PPARα agonists increase beta-oxidation and glycerol kinase expression in human white adipocytes (56, 57). PPARα has also been shown to induce subcutaneous white adipose tissue browning and stimulate thermogenesis, improve insulin resistance, and reduce inflammation in animal models when treated with fenofibrate (58). However, it is important to note that results obtained in animal models may not directly translate to humans due to species differences (55).

## 2 Physical exercise drives several mechanisms aimed at improved usage of energy substrates

### 2.1 Physical exercise as part of the interventions aimed at MASLD regression

Aerobic exercise, such as walking and cycling, is a cost-effective and widely accessible non-pharmacological intervention for the general public. It is characterized by increased energy expenditure during exercise sessions and has been shown to have positive effects on various factors associated with MASLD, including hemoglobin A1c, resting blood pressure, and serum cholesterol levels (59). Nonetheless, it is important to note that aerobic exercise can lead to fatigue and discomfort, potentially compromising long-term adherence to exercise regimens.

Several informative reviews have been published in the medical literature, discussing the role of exercise prescription for MASLD and highlighting the potential benefits of both aerobic and resistance exercises (60–64). However, there remains a lack of clarity regarding the optimal exercise protocol, including the recommended frequency, intensity, and duration of aerobic and resistance exercises for improving MASLD. Furthermore, considering the high prevalence of cardiovascular diseases in individuals with MASLD (65, 66), a comparison of exercise types based on energy consumption has yet to be conducted. Additionally, it is important to target the pathogenesis of MASLD, specifically insulin resistance and decreased metabolic flexibility, through physical exercise as potential mechanisms for mitigating liver damage. In the following paragraphs, we will analyze the available evidence to better understand the benefits of aerobic and resistance exercise in relation to the metabolic changes they elicit.

### 2.2 Frequency, intensity, and duration of exercise for improving hepatic steatosis in MASLD

In a comprehensive review of clinical trials, Hashida et al. included twenty-four exercise protocols from 18 articles, demonstrating a decrease in hepatic steatosis in 91.7% of the protocols (64). The median age of patients was 48 years, with a median BMI of 30.9 kg/m^2^. All protocols had a frequency of exercise of 3 times per week. Moderate-intensity aerobic exercise for a duration of 40 min over a 12-week period was the typical protocol. Aerobic exercise led to improvements in BMI and serum alanine aminotransferase levels.

Most protocols focused on conventional aerobic exercises, such as walking or cycling at a constant intensity. However, a study on high-intensity interval training (HIIT) demonstrated reductions in whole-body fat mass, serum alanine aminotransferase levels, and hepatic lipids. HIIT involved high-intensity exercise intervals followed by recovery periods (67).

Also, very short or low-intensity exercises may be insufficient for improving hepatic steatosis, emphasizing the importance of exercise energy consumption (68, 69).

Aerobic exercise is known to promote lipolysis in adipose tissues, leading to the production of acetyl-CoA through increased beta-oxidation (70). Acetyl-CoA is then metabolized in the tricarboxylic acid cycle, resulting in ATP production in the mitochondria’s electron transport system. Aerobic exercise also upregulates certain proteins, such as uncoupling protein-1 and peroxisome proliferator-activated receptor gamma (71), which further enhance lipolysis in adipose tissues (72). Studies have shown that aerobic exercise can decrease serum levels of resistin and increase levels of high molecular weight adiponectin, indicating potential benefits for individuals with conditions like hypertension and MASLD (73–75).

### 2.3 Resistance exercise for improving hepatic steatosis in MASLD

In the same work from Hashida et al. (64) resistance exercise resulted overall beneficial in MASLD. Based on seven protocols from seven studies, a reduction in hepatic steatosis was observed in 85.7% of the protocols, including five randomized controlled trials (76–82). The median age of patients was 49.2 years, with a median BMI of 30.6 kg/m^2^. The exercise frequency was consistently three times per week, with a median metabolic equivalent (METs) of 3.5, exercise duration of 45 min, and a 12-week period.

Resistance exercise resulted in modest changes in BMI and serum alanine aminotransferase (ALT) levels. Three protocols demonstrated improvements in hepatic steatosis without significant weight loss (76, 80, 83). MR spectroscopy showed a decrease in intrahepatic lipid levels in three studies (13%; 3%; and 25% reduction from baseline (76–78)).

While most studies used weight machines, one study with 53 individuals who trained for 12 weeks evaluated the effects of simple bodyweight resistance exercises such as push-ups and squats, demonstrating improvements in muscle mass, ALT levels, and hepatic steatosis (83).

One study did not show improvements in hepatic steatosis through resistance exercise (82). The longer duration (32 weeks) and significant increase in body weight in that study may have influenced the results.

The optimal duration for resistance exercise to improve hepatic steatosis appears to be 12 weeks. General recommendations include three sets of 8–12 repetitions, three times per week, targeting major muscle groups. Using a variety of weight training exercises is recommended.

Resistance exercise may have distinct therapeutic characteristics compared to aerobic exercise in MASLD patients, as it showed modest changes in BMI and demonstrated steatosis improvement independent of body weight reduction.

Further research is needed to explore the long-term effects of resistance exercise and to better understand its mechanisms in improving hepatic steatosis.

Resistance exercise offers benefits for hepatic steatosis with less energy consumption. The exact mechanisms behind these benefits are not fully understood, but it may involve muscle fiber type-specific adaptations. Muscle fibers can be categorized into type I (slow oxidative) and type II (fast glycolytic) based on their energy metabolism. Resistance exercise has been found to specifically promote hypertrophy in type II muscle fibers, while not significantly affecting type I fibers. Additionally, resistance exercise has been shown to increase the expression of GLUT-4 in type II fibers and enhance intracellular insulin sensitivity (84–87). These changes may contribute to improvements in hepatic steatosis and insulin resistance.

Moreover, resistance exercise may improve MASLD through muscle-liver crosstalk mediated by a myokine called irisin (88, 89). Irisin is released by skeletal muscles and has been shown to increase thermogenesis and energy expenditure by promoting the browning of subcutaneous adipocytes. It also exhibits regulatory effects on lipid metabolism in hepatocytes (88).

Studies have demonstrated that recombinant irisin inhibits the expression of key regulators of lipogenesis, such as sterol regulatory element-binding protein-1c, and lipogenic enzymes in hepatocytes (90). Overexpression of irisin has been found to improve hepatic steatosis in obese mice. Interestingly, individuals with MASLD have been reported to have lower serum irisin levels compared to healthy individuals (91).

Recent research by Kim et al. investigated the effects of aerobic and resistance exercises on circulating irisin levels. They found that resistance exercise significantly increased circulating irisin levels, while aerobic exercise did not produce the same effect. This suggests that resistance exercise specifically influences lipid metabolism in the liver through the action of irisin (92).

**Figure 1:**
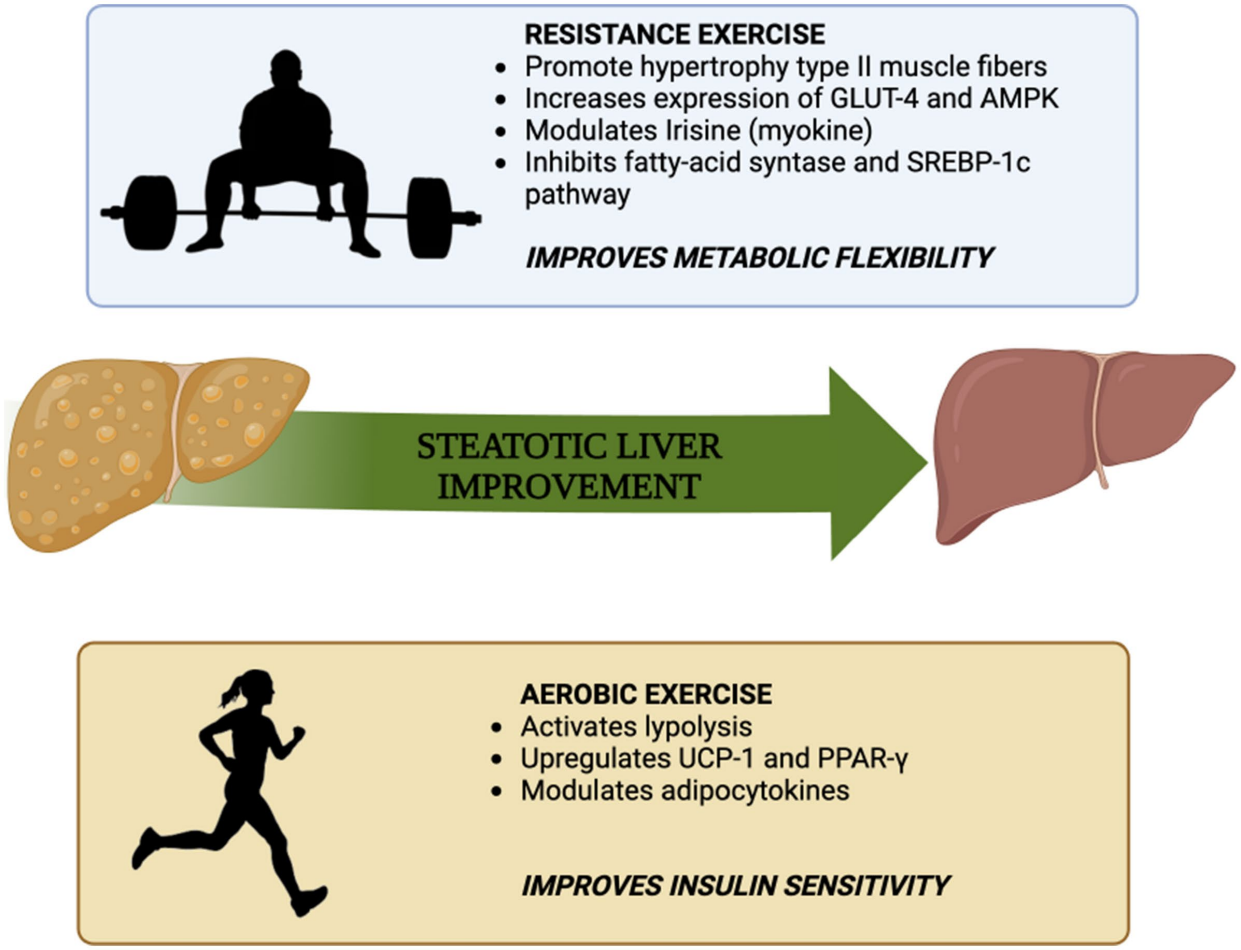
Summary of the mechanisms by which aerobic exercise and resistance exercise improve insulin sensitivity and metabolic flexibility, respectively [adapted from Hashida et al. (64)].

Therefore, one additional explanation for the improvement of MASLD through resistance exercise, despite lower energy consumption compared to aerobic exercise, could be attributed to the muscle-liver crosstalk facilitated by irisin. These mechanisms are key players in the switch from impaired metabolic flexibility to enhanced metabolic flexibility and might be responsible for pleiotropic, and not only liver-focused, benefits of resistance exercise. In details, reverting the futile cycle of *de novo lipogenesis* and beta-oxidation that is typical of decreased metabolic flexibility, may improve the oxidation of glucose and foster the flux of energetic intermediates of lipid metabolism toward mitochondrial oxidation therefore accelerating the de-fatting of the liver (see Figure 1).

### 2.4 Skeletal muscle contractions enhance glucose uptake and by-passes insulin resistance

During resistance exercise, muscle contractions activate several molecular signaling pathways that influence glucose uptake. One key pathway involves the activation of AMPK. AMPK activation stimulates glucose uptake by promoting the translocation of glucose transporter GLUT4 to the cell surface, thereby enhancing the uptake of glucose into muscle cells (93).

The mechanisms underlying this increase are complex, involving the docking and fusion of vesicles containing GLUT4 with the cellular surface membrane. It has been demonstrated that muscular contraction stimulates the vesicle-associated SNARE proteins, facilitating the merging of GLUT4-containing vesicles with the cellular surface membrane (94).

Additionally, resistance exercise activates the insulin signaling pathway, which plays a crucial role in glucose uptake. Insulin, released by the pancreas in response to elevated blood glucose levels, binds to insulin receptors on muscle cells, leading to downstream signaling events that result in the translocation of GLUT4 to the cell surface. This allows for increased glucose uptake by skeletal muscle cells (94).

**Figure 2:**
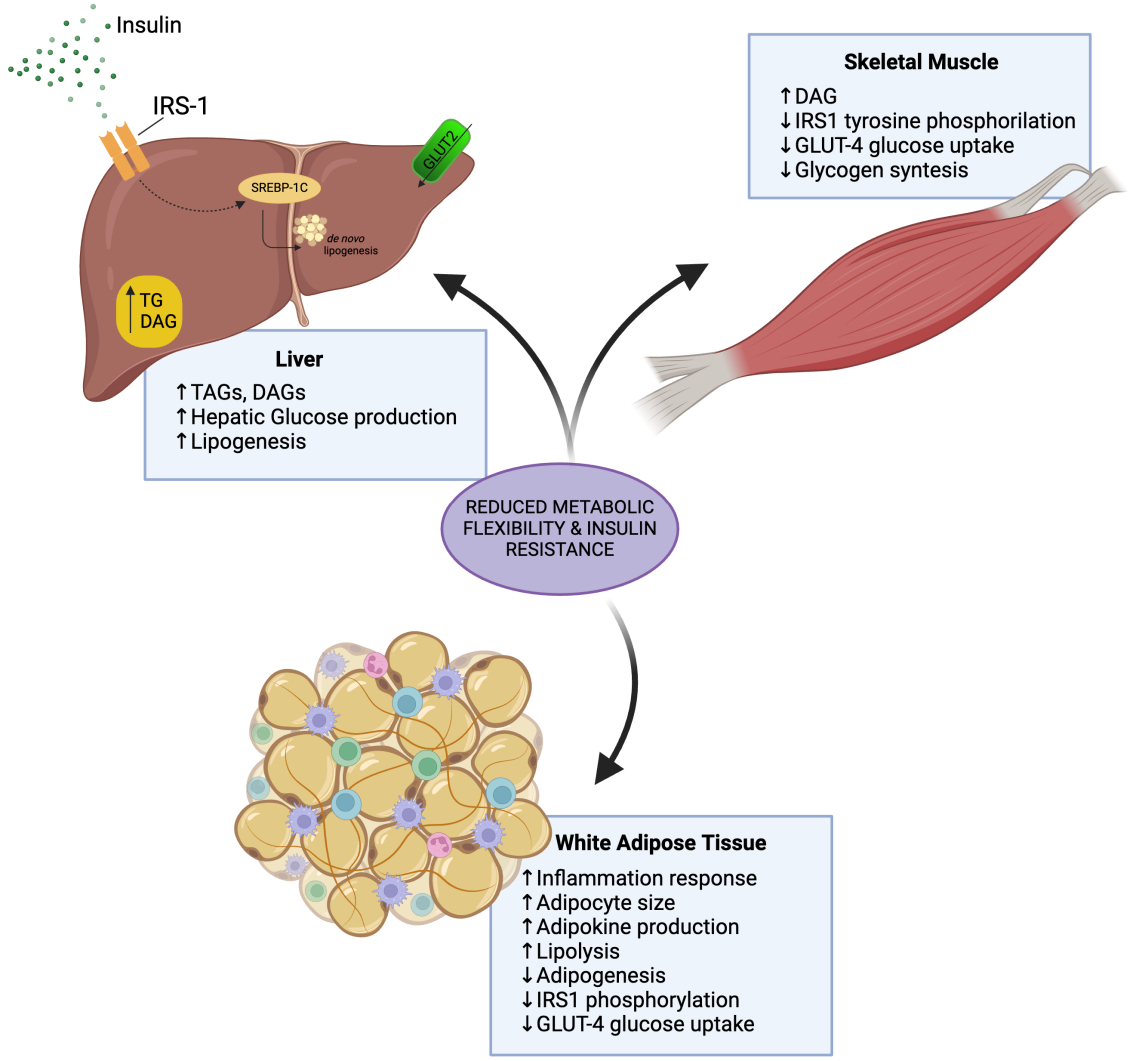
Effects of insulin resistance in the liver, white adipose tissue, and skeletal muscle.

Overall, increased blood glucose clearance and increased liver fat mobility derived from resistance exercise may exert a positive effect in liver health preventing and reverting MASLD progression (Figure 2).

## 3 Dietary components of Mediterranean diet can act as promoter of insulin sensitivity

Certain dietary components, such as specific nutrients and dietary patterns, have been associated with improvements in insulin sensitivity. Examining the evidence surrounding these components enhances our understanding of how dietary interventions may influence insulin resistance in the context of MASLD.

Contemporary literature is focusing on evaluating dietary patterns aiming to comprehend the collective impact of the entire diet on specific health conditions. This is in contrast to the focus of the past studies which were more aimed to individualize the role of specific nutrients on health.

A systematic review conducted by Tanase et al. (95) explores the intricate relationship among T2D, insulin resistance (IR), and MASLD. The review compared nine different dietary patterns, including vegetarian, Mediterranean diet, high-protein diet, moderate-carbohydrate diet, low-carbohydrate diet, low-glycaemic index/GL diet, paleolithic diet, low-fat diet, and a control diet. More specifically, the review revealed that, while all dietary patterns induced a significant reduction in Hb1Ac levels after 12 weeks, the traditional Mediterranean diet emerged as the most efficacious in improving postprandial hyperglycaemia and insulin sensitivity at an equivalent body weight. This underscores the role of dietary quality in enhancing these metabolic parameters.

The impact of the Mediterranean diet on insulin resistance stems from the synergistic interplay of its constituent elements, characterized by elevated consumption of olive oil, nuts, fruits, legumes, vegetables, and fish, combined with reduced intake of red meat, processed meats, and sweets. Notably, mono-and polyunsaturated fatty acids (MUFAs and PUFAs), primarily sourced from nuts and olive oil within this dietary pattern, gained a lot of interest through the years. A particular significance were acquired by omega-3 PUFAs, specifically eicosapentaenoic acid (EPA) and docosahexaenoic acid (DHA), which are renowned for their antioxidant and anti-inflammatory properties (95–97).

Moreover, these fatty acids have demonstrated efficacy in enhancing insulin sensitivity, ameliorating steatohepatitis, and reducing intrahepatic triglyceride content. The mechanistic underpinnings involve the downregulation of SREBP-1c and activation of PPARα (98).

PUFAs also play a significant influence on glucose metabolism by binding and stimulating G-protein-coupled receptors (GPCRs), such as GPR120. This stimulation leads to an increased secretion GLP-1 by enteroendocrine L cells. GLP-1 stimulates insulin release from pancreatic β-cells, enhances glucose uptake in skeletal muscles, and may modulate satiety perception by attenuating appetite through central nervous system actions. A noteworthy observation from the review is that the favorable impact of unsaturated fatty acids on insulin sensitivity over saturated fatty acids is evident when total fat intake is below 37% of energy, while higher fat intake increases the risk of insulin resistance irrespective of fat quality (99).

Focusing on the carbohydrate sources prevalent in the Mediterranean diet, these primarily consist of legumes and whole grains. These components exhibit efficacy in postprandial hyperglycaemia, which increase more gradually over time, and enhance insulin sensitivity. The beneficial effects can be attributed to the high fibre content, especially soluble fibre. Soluble fibre attenuates the pronounced glycaemic peaks characteristic of a Western diet, thereby mitigating oxidative stress, preserving pancreatic beta cell integrity, delaying gastric emptying, and correlating with reduced insulin requirements (100).

Furthermore, the Mediterranean diet is distinguished by its elevated consumption of flavonoids derived from vegetables, nuts, and whole grains. A review reported that individuals with heightened flavonoid intake exhibited an 11% reduced risk of developing T2D during follow-up, a condition frequently associated with MASLD (99).

It is essential to note that the Mediterranean diet, as a dietary pattern, not only incorporates elements conducive to improving insulin sensitivity but also entails a reduction in the consumption of sugar-rich foods, particularly added sugars such as fructose. The latter has been linked to the development of MASLD and insulin resistance, attributed to increased hepatic lipogenesis, alterations in intestinal microflora, heightened intestinal permeability, endotoxemia, elevated hepatic Tumor Necrosis Factor-α production, and lipid peroxidation (79).

With regards to other dietary patterns investigated in the existing literature, the majority of studies fail to independently explore the impact of dietary quality aside from weight loss—a critical factor in enhancing insulin sensitivity. Consequently, there is a lack of consistent evidence demonstrating the direct role of these patterns in insulin sensitivity under conditions of normocaloric diets.

Notably, irrespective of dietary quality, weight loss has consistently demonstrated improvements in postprandial hyperglycaemia and insulin resistance. This is evidenced by enhanced serum liver enzymes, reduced hepatic lipid accumulation, and alleviation of liver inflammation following weight loss interventions (100). This is also supported by the joint EASL-EASD-EASO European Clinical Practice Guidelines for the Management of MASLD (101).

Moreover, a crossover randomized controlled trial sought to assess the impact of distinct dietary patterns on insulin sensitivity within the context of hepatic steatosis. The study involved twelve non-diabetic subjects (6 females/6 males) with biopsy-proven MASLD, subject to a randomized, cross-over 6-week dietary intervention. The dietary patterns examined were the Mediterranean diet and a low-fat, high-carbohydrate diet (LF/HCD) as a control. Notably, the findings demonstrated that, even in the absence of weight loss, adherence to the Mediterranean diet led to a reduction in hepatic steatosis and an improvement in insulin sensitivity (102).

## 4 Resistance exercise can foster the switch from impaired metabolic flexibility to enhanced flexibility through activation of AMPK while aerobic exercise promotes insulin sensitivity

Exercise induces acute changes in the molecular and biochemical processes involved in mobilizing energy from carbohydrates and fatty acids, leading to an enhanced energy supply. AMPK, a key regulator of energy sensing, is stimulated by regular physical activity (103). Drug-induced activation of AMPK produces gene expression patterns similar to those observed during exercise (104).

In response to resistance exercise, healthy individuals can efficiently switch between glucose and fatty acid oxidation for energy production, depending on exercise intensity and duration. As exercise intensity increases, there is a greater reliance on glucose oxidation through oxidative phosphorylation, while anaerobic glycolysis becomes the predominant pathway during higher intensity exercise (105). These metabolic shifts occur independently of insulin, as circulating insulin levels remain suppressed during exercise, and the contribution of fatty acid oxidation to energy production decreases proportionally (106, 107).

During acute exercise, the release of free fatty acids (FFAs) from adipose tissue is primarily driven by increased levels of catecholamines, with abdominal subcutaneous white adipose tissue (WAT) being the main source (108). In contrast, visceral adipose tissue plays a minor role due to its smaller size, particularly in healthy metabolic states (108).

On the other hand, the increased energy demand during aerobic exercise can upregulate the activity of PPAR-α within muscle cells. As discussed in the section 1.7, high PPARα activity in skeletal muscle up-regulates the delivery of fatty acids from WAT to skeletal muscle.

Studies blocking β-adrenergic receptors in abdominal subcutaneous WAT have demonstrated the role of WAT in metabolic flexibility during exercise by reducing lipolysis (109). Furthermore, the deletion of adipose triglyceride lipase (ATGL), a key enzyme involved in lipolysis, has been shown to impair exercise performance in a mouse model due to a decreased supply of FFAs to skeletal muscle (110).

Obesity may lead to a decrease in β2-adrenergic receptor density in adipocytes, resulting in resistance to catecholamine action and a blunted release of FFAs from WAT during fasting and exercise. The capacity of WAT to mobilize FFAs during acute exercise and in response to chronic training plays a crucial role in facilitating overall fat oxidation, particularly within skeletal muscle (111). Resistance physical activity is therefore essential for increasing energy supply to tissues and improving metabolic flexibility. At the same time, aerobic exercise can improve insulin resistance mainly through the re-arrangement of FFAs from WAT to tissues on a state of intense mitochondrial lipids oxidation. Ultimately, both kind of training, through different mechanisms contribute to improvement of steatotic liver.

Metabolic flexibility encompasses a diverse array of pathways and mechanisms related to fuel selection, energy expenditure, and overall metabolic function. These aspects present potential targets for therapeutic interventions, particularly in the context of MASLD. Conflicting data have emerged regarding the impact of increasing mitochondrial FFA flux and oxidation on insulin resistance (112, 113). Notably, these strategies do not replicate the same energy expenditure or demand observed during exercise. Consequently, while interventions aimed at modifying substrate metabolism or metabolic flexibility may hold implications for obesity and metabolic diseases associated with nutrient overload, they cannot be considered genuine exercise mimetics unless accompanied by an elevated energy demand (12).

## 5 Conclusion

Impaired metabolic flexibility impacts the body ability to properly adjust to different physiological conditions, including exercise. Insulin resistance involves various tissues and organs, with the liver being a key organ where the shift from metabolic flexibility to inflexibility occurs. During exercise and under other changing conditions, decreased metabolic flexibility can compromise the body ability to handle different substrates.

Restoring metabolic flexibility and reducing insulin resistance are important targets for improving health outcomes. Exercise, combined with dietary and pharmacological interventions, may be an effective way to help restore metabolic flexibility and mitigate the liver damage driven by insulin resistance.

## References

[1] Younossi ZM Henry L. Epidemiology of non-alcoholic fatty liver disease and hepatocellular carcinoma. JHEP Rep. (2021) 3:100305. doi: 10.1016/j.jhepr.2021.100305, PMID: 34189448 PMC8215299

[2] Colosimo S Marchesini G. Editorial: should NAFLD be included in the definition of metabolic syndrome? Aliment Pharmacol Ther. (2023) 57:1151–2. doi: 10.1111/apt.17411 37094320

[3] Marjot T Moolla A Cobbold JF Hodson L Tomlinson JW. Nonalcoholic fatty liver disease in adults: current concepts in etiology, outcomes, and management. Endocr Rev. (2020) 41:66–117. doi: 10.1210/endrev/bnz009, PMID: 31629366

[4] Colosimo S Ravaioli F Petroni ML Brodosi L Marchignoli F Barbanti FA. Effects of antidiabetic agents on steatosis and fibrosis biomarkers in type 2 diabetes: a real-world data analysis. Liver Int. (2021) 41:731–42. doi: 10.1111/liv.14799, PMID: 33497019 PMC8248247

[5] ColosimoSTanGDPetroniMLMarchesiniGTomlinsonJW. Improved glycaemic control in patients with type 2 diabetes has a beneficial impact on NAFLD, independent of change in BMI or glucose lowering agent. Nutr Metab Cardiovasc Dis. (2022) 33:640–8. doi: 10.1016/j.numecd.2022.12.01036710114

[6] Vilar-Gomez E Martinez-Perez Y Calzadilla-Bertot L Torres-Gonzalez A Gra-Oramas B Gonzalez-Fabian L. Weight loss through lifestyle modification significantly reduces features of nonalcoholic steatohepatitis. Gastroenterology. (2015) 149:367–378.e5. doi: 10.1053/j.gastro.2015.04.005, PMID: 25865049

[7] Friedman SL Neuschwander-Tetri BA Rinella M Sanyal AJ. Mechanisms of NAFLD development and therapeutic strategies. Nat Med. (2018) 24:908–22. doi: 10.1038/s41591-018-0104-9, PMID: 29967350 PMC6553468

[8] Bischoff SC Bernal W Dasarathy S Merli M Plank LD Schütz T. ESPEN practical guideline: clinical nutrition in liver disease. Clin Nutr. (2020) 39:3533–62. doi: 10.1016/j.clnu.2020.09.001, PMID: 33213977

[9] Harrison SA Bedossa P Guy CD Schattenberg JM Loomba R Taub R. A phase 3, randomized, controlled trial of Resmetirom in NASH with liver fibrosis. N Engl J Med. (2024) 390:497–509. doi: 10.1056/NEJMoa2309000, PMID: 38324483

[10] Kokkorakis M Boutari C Hill MA Kotsis V Loomba R Sanyal AJ. Resmetirom, the first approved drug for the management of metabolic dysfunction-associated steatohepatitis: trials, opportunities, and challenges. Metabolism. (2024) 154:155835. doi: 10.1016/j.metabol.2024.155835, PMID: 38508373

[11] Bugianesi E McCullough AJ Marchesini G. Insulin resistance: a metabolic pathway to chronic liver disease. Hepatology. (2005) 42:987–1000. doi: 10.1002/hep.20920 16250043

[12] Colosimo S Mitra SK Chaudhury T Marchesini G. Insulin resistance and metabolic flexibility as drivers of liver and cardiac disease in T2DM. Diabetes Res Clin Pract. (2023) 206:111016. doi: 10.1016/j.diabres.2023.111016, PMID: 37979728

[13] Carneiro IP Elliott SA Siervo M Padwal R Bertoli S Battezzati A. Is obesity associated with altered energy expenditure? Adv Nutr. (2016) 7:476–87. doi: 10.3945/an.115.008755, PMID: 27184275 PMC4863259

[14] Maciak S Sawicka D Sadowska A Prokopiuk S Buczyńska S Bartoszewicz M. Low basal metabolic rate as a risk factor for development of insulin resistance and type 2 diabetes. BMJ Open Diabetes Res Care. (2020) 8:e001381. doi: 10.1136/bmjdrc-2020-001381, PMID: 32690630 PMC7373309

[15] Sampath Kumar A Arun Maiya G Shastry BA Vaishali K Maiya S Umakanth S. Correlation between basal metabolic rate, visceral fat and insulin resistance among type 2 diabetes mellitus with peripheral neuropathy. Diabetes Metab Syndr. (2019) 13:344–8. doi: 10.1016/j.dsx.2018.10.005, PMID: 30641723

[16] Blasco RR. Resting energy expenditure; assessment methods and applications. Nutr Hosp. (2015) 31:245–54. 25719792 10.3305/nh.2015.31.sup3.8772

[17] Astrup A Gøtzsche PC van de Werken K Ranneries C Toubro S Raben A. Meta-analysis of resting metabolic rate in formerly obese subjects. Am J Clin Nutr. (1999) 69:1117–22. doi: 10.1093/ajcn/69.6.1117, PMID: 10357728

[18] Martínez-Gómez MG Roberts BM. Metabolic adaptations to weight loss: a brief review. J Strength Cond Res. (2022) 36:2970–81. doi: 10.1519/JSC.0000000000003991, PMID: 33677461

[19] Marchesini G Cuzzolaro M Mannucci E Dalle Grave R Gennaro M Tomasi F. Weight cycling in treatment-seeking obese persons: data from the QUOVADIS study. Int J Obes Relat Metab Disord. (2004) 28:1456–62. doi: 10.1038/sj.ijo.0802741, PMID: 15314631

[20] Hall KD Guo J. Obesity energetics: body weight regulation and the effects of diet composition. Gastroenterology. (2017) 152:1718–27.e3. doi: 10.1053/j.gastro.2017.01.052, PMID: 28193517 PMC5568065

[21] Bi X Forde CG Goh AT Henry CJ. Basal metabolic rate and body composition predict habitual food and macronutrient intakes: gender differences. Nutrients. (2019) 11:2653. doi: 10.3390/nu11112653, PMID: 31689964 PMC6893862

[22] Stiegler P Cunliffe A. The role of diet and exercise for the maintenance of fat-free mass and resting metabolic rate during weight loss. Sports Med. (2006) 36:239–62. doi: 10.2165/00007256-200636030-00005, PMID: 16526835

[23] Johnstone AM Murison SD Duncan JS Rance KA Speakman JR. Factors influencing variation in basal metabolic rate include fat-free mass, fat mass, age, and circulating thyroxine but not sex, circulating leptin, or triiodothyronine. Am J Clin Nutr. (2005) 82:941–8. doi: 10.1093/ajcn/82.5.941 16280423

[24] Smith RL Soeters MR Wüst RCI Houtkooper RH. Metabolic flexibility as an adaptation to energy resources and requirements in health and disease. Endocr Rev. (2018) 39:489–517. doi: 10.1210/er.2017-00211, PMID: 29697773 PMC6093334

[25] Codogno P Meijer AJ. Autophagy: a potential link between obesity and insulin resistance. Cell Metab. (2010) 11:449–51. doi: 10.1016/j.cmet.2010.05.006, PMID: 20519116

[26] Schultze SM Hemmings BA Niessen M Tschopp O. PI3K/AKT, MAPK and AMPK signalling: protein kinases in glucose homeostasis. Expert Rev Mol Med. (2012) 14:e1. doi: 10.1017/S1462399411002109, PMID: 22233681

[27] Sandoval DA D'Alessio DA. Physiology of proglucagon peptides: role of glucagon and GLP-1 in health and disease. Physiol Rev. (2015) 95:513–48. doi: 10.1152/physrev.00013.2014 25834231

[28] Gastaldelli A Gaggini M DeFronzo R. Glucose kinetics: an update and novel insights into its regulation by glucagon and GLP-1. Curr Opin Clin Nutr Metab Care. (2017) 20:300–9. doi: 10.1097/MCO.0000000000000384 28463898

[29] Röder PV Wu B Liu Y Han W. Pancreatic regulation of glucose homeostasis. Exp Mol Med. (2016) 48:e219. doi: 10.1038/emm.2016.6, PMID: 26964835 PMC4892884

[30] García-Ruiz C Baulies A Mari M García-Rovés PM Fernandez-Checa JC. Mitochondrial dysfunction in non-alcoholic fatty liver disease and insulin resistance: cause or consequence? Free Radic Res. (2013) 47:854–68. doi: 10.3109/10715762.2013.830717, PMID: 23915028

[31] Samuel VT Shulman GI. Mechanisms for insulin resistance: common threads and missing links. Cell. (2012) 148:852–71. doi: 10.1016/j.cell.2012.02.017, PMID: 22385956 PMC3294420

[32] Liang H Ward WF. PGC-1alpha: a key regulator of energy metabolism. Adv Physiol Educ. (2006) 30:145–51. doi: 10.1152/advan.00052.2006 17108241

[33] Schreiber SN Emter R Hock MB Knutti D Cardenas J Podvinec M. The estrogen-related receptor alpha (ERRalpha) functions in PPARgamma coactivator 1alpha (PGC-1alpha)-induced mitochondrial biogenesis. Proc Natl Acad Sci USA. (2004) 101:6472–7. doi: 10.1073/pnas.0308686101, PMID: 15087503 PMC404069

[34] Piccinin E Villani G Moschetta A. Metabolic aspects in NAFLD, NASH and hepatocellular carcinoma: the role of PGC1 coactivators. Nat Rev Gastroenterol Hepatol. (2019) 16:160–74. doi: 10.1038/s41575-018-0089-3, PMID: 30518830

[35] Sánchez-Ramos C Prieto I Tierrez A Laso J Valdecantos MP Bartrons R. PGC-1α downregulation in Steatotic liver enhances ischemia-reperfusion injury and impairs ischemic preconditioning. Antioxid Redox Signal. (2017) 27:1332–46. doi: 10.1089/ars.2016.6836, PMID: 28269997

[36] Morris EM Meers GM Booth FW Fritsche KL Hardin CD Thyfault JP. PGC-1α overexpression results in increased hepatic fatty acid oxidation with reduced triacylglycerol accumulation and secretion. Am J Physiol Gastrointest Liver Physiol. (2012) 303:G979–92. doi: 10.1152/ajpgi.00169.2012, PMID: 22899824 PMC3469696

[37] Shulman GI. Ectopic fat in insulin resistance, dyslipidemia, and cardiometabolic disease. N Engl J Med. (2014) 371:1131–41. doi: 10.1056/NEJMra1011035, PMID: 25229917

[38] Pessayre D. Role of mitochondria in non-alcoholic fatty liver disease. J Gastroenterol Hepatol. (2007) 22:S20–7. doi: 10.1111/j.1440-1746.2006.04640.x 17567459

[39] Barrès R Osler ME Yan J Rune A Fritz T Caidahl K. Non-CpG methylation of the PGC-1alpha promoter through DNMT3B controls mitochondrial density. Cell Metab. (2009) 10:189–98. doi: 10.1016/j.cmet.2009.07.011, PMID: 19723495

[40] Malin SK Kirwan JP Sia CL González F. Glucose-stimulated oxidative stress in mononuclear cells is related to pancreatic β-cell dysfunction in polycystic ovary syndrome. J Clin Endocrinol Metab. (2014) 99:322–9. doi: 10.1210/jc.2013-3177, PMID: 24203060 PMC3879676

[41] Chadt A Immisch A de Wendt C Springer C Zhou Z Stermann T. Deletion of both Rab-GTPase-activating proteins TBC14KO and TBC1D4 in mice eliminates insulin-and AICAR-stimulated glucose transport. Diabetes. (2015) 64:746–59. doi: 10.2337/db15-er04 25249576

[42] Souza-Tavares H Miranda CS Vasques-Monteiro IML Sandoval C Santana-Oliveira DA Silva-Veiga FM. Peroxisome proliferator-activated receptors as targets to treat metabolic diseases: focus on the adipose tissue, liver, and pancreas. World J Gastroenterol. (2023) 29:4136–55. doi: 10.3748/wjg.v29.i26.4136, PMID: 37475842 PMC10354577

[43] Kong S Cai B Nie Q. PGC-1α affects skeletal muscle and adipose tissue development by regulating mitochondrial biogenesis. Mol Gen Genomics. (2022) 297:621–33. doi: 10.1007/s00438-022-01878-2, PMID: 35290519

[44] Argentato PP de Cássia CH Estadella D Pisani LP. Programming mediated by fatty acids affects uncoupling protein 1 (UCP-1) in brown adipose tissue. Br J Nutr. (2018) 120:619–27. doi: 10.1017/S0007114518001629, PMID: 30176958

[45] Hammarstedt A Jansson PA Wesslau C Yang X Smith U. Reduced expression of PGC-1 and insulin-signaling molecules in adipose tissue is associated with insulin resistance. Biochem Biophys Res Commun. (2003) 301:578–82. doi: 10.1016/S0006-291X(03)00014-7, PMID: 12565902

[46] Kleiner S Mepani RJ Laznik D Ye L Jurczak MJ Jornayvaz FR. Development of insulin resistance in mice lacking PGC-1α in adipose tissues. Proc Natl Acad Sci USA. (2012) 109:9635–40. doi: 10.1073/pnas.1207287109, PMID: 22645355 PMC3386123

[47] Schupp M Lazar MA. Fingered for a fat fate. Cell Metab. (2010) 11:244–5. doi: 10.1016/j.cmet.2010.02.014, PMID: 20374953

[48] Wahli W Michalik L. PPARs at the crossroads of lipid signaling and inflammation. Trends Endocrinol Metab. (2012) 23:351–63. doi: 10.1016/j.tem.2012.05.001, PMID: 22704720

[49] Islinger M Grille S Fahimi HD Schrader M. The peroxisome: an update on mysteries. Histochem Cell Biol. (2012) 137:547–74. doi: 10.1007/s00418-012-0941-4, PMID: 22415027

[50] Bougarne N Weyers B Desmet SJ Deckers J Ray DW Staels B. Molecular actions of PPARα in lipid metabolism and inflammation. Endocr Rev. (2018) 39:760–802. doi: 10.1210/er.2018-00064, PMID: 30020428

[51] Vu-Dac N Schoonjans K Laine B Fruchart JC Auwerx J Staels B. Negative regulation of the human apolipoprotein A-I promoter by fibrates can be attenuated by the interaction of the peroxisome proliferator-activated receptor with its response element. J Biol Chem. (1994) 269:31012–8. doi: 10.1016/S0021-9258(18)47383-8, PMID: 7983038

[52] Francque S Szabo G Abdelmalek MF Byrne CD Cusi K Dufour JF. Nonalcoholic steatohepatitis: the role of peroxisome proliferator-activated receptors. Nat Rev Gastroenterol Hepatol. (2021) 18:24–39. doi: 10.1038/s41575-020-00366-5 33093663

[53] Lim WS Ng DL Kor SB Wong HK Tengku-Muhammad TS Choo QC. Tumour necrosis factor alpha down-regulates the expression of peroxisome proliferator activated receptor alpha (PPARα) in human hepatocarcinoma Hep G2 cells by activation of NF-κB pathway. Cytokine. (2013) 61:266–74. doi: 10.1016/j.cyto.2012.10.007, PMID: 23141142

[54] Li P Zhu Z Lu Y Granneman JG. Metabolic and cellular plasticity in white adipose tissue II: role of peroxisome proliferator-activated receptor-alpha. Am J Physiol Endocrinol Metab. (2005) 289:E617–26. doi: 10.1152/ajpendo.00010.2005, PMID: 15941786

[55] Ribet C Montastier E Valle C Bezaire V Mazzucotelli A Mairal A. Peroxisome proliferator-activated receptor-alpha control of lipid and glucose metabolism in human white adipocytes. Endocrinology. (2010) 151:123–33. doi: 10.1210/en.2009-0726, PMID: 19887568

[56] Bogacka I Xie H Bray GA Smith SR. Pioglitazone induces mitochondrial biogenesis in human subcutaneous adipose tissue in vivo. Diabetes. (2005) 54:1392–9. doi: 10.2337/diabetes.54.5.1392, PMID: 15855325

[57] Mazzucotelli A Viguerie N Tiraby C Annicotte JS Mairal A Klimcakova E. The transcriptional coactivator peroxisome proliferator activated receptor (PPAR) gamma coactivator-1 alpha and the nuclear receptor PPAR alpha control the expression of glycerol kinase and metabolism genes independently of PPAR gamma activation in human white adipocytes. Diabetes. (2007) 56:2467–75. doi: 10.2337/db06-1465 17646210

[58] Rachid TL Penna-de-Carvalho A Bringhenti I Aguila MB Mandarim-de-Lacerda CA Souza-Mello V. PPAR-α agonist elicits metabolically active brown adipocytes and weight loss in diet-induced obese mice. Cell Biochem Funct. (2015) 33:249–56. doi: 10.1002/cbf.3111, PMID: 25959716

[59] Kelley GA Kelley KS. Efficacy of aerobic exercise on coronary heart disease risk factors. Prev Cardiol. (2008) 11:71–5. doi: 10.1111/j.1751-7141.2008.08037.x 18401233

[60] Keating SE Hackett DA George J Johnson NA. Exercise and non-alcoholic fatty liver disease: a systematic review and meta-analysis. J Hepatol. (2012) 57:157–66. doi: 10.1016/j.jhep.2012.02.023 22414768

[61] Berzigotti A Saran U Dufour JF. Physical activity and liver diseases. Hepatology. (2016) 63:1026–40. doi: 10.1002/hep.28132 26313307

[62] Johnson NA Keating SE George J. Exercise and the liver: implications for therapy in fatty liver disorders. Semin Liver Dis. (2012) 32:65–79. doi: 10.1055/s-0032-1306427, PMID: 22418889

[63] Marchesini G Petta S Dalle GR. Diet, weight loss, and liver health in nonalcoholic fatty liver disease: pathophysiology, evidence, and practice. Hepatology. (2016) 63:2032–43. doi: 10.1002/hep.28392, PMID: 26663351

[64] Hashida R Kawaguchi T Bekki M Omoto M Matsuse H Nago T. Aerobic vs. resistance exercise in non-alcoholic fatty liver disease: a systematic review. J Hepatol. (2017) 66:142–52. doi: 10.1016/j.jhep.2016.08.023, PMID: 27639843

[65] Ghouri N Preiss D Sattar N. Liver enzymes, nonalcoholic fatty liver disease, and incident cardiovascular disease: a narrative review and clinical perspective of prospective data. Hepatology. (2010) 52:1156–61. doi: 10.1002/hep.23789, PMID: 20658466

[66] Targher G Byrne CD Lonardo A Zoppini G Barbui C. Non-alcoholic fatty liver disease and risk of incident cardiovascular disease: a meta-analysis. J Hepatol. (2016) 65:589–600. doi: 10.1016/j.jhep.2016.05.013 27212244

[67] Hallsworth K Thoma C Hollingsworth KG Cassidy S Anstee QM Day CP. Modified high-intensity interval training reduces liver fat and improves cardiac function in non-alcoholic fatty liver disease: a randomized controlled trial. Clin Sci (Lond). (2015) 129:1097–105. doi: 10.1042/CS20150308, PMID: 26265792

[68] Fealy CE Haus JM Solomon TP Pagadala M Flask CA McCullough AJ. Short-term exercise reduces markers of hepatocyte apoptosis in nonalcoholic fatty liver disease. J Appl Physiol (1985). (2012) 113:1–6. doi: 10.1152/japplphysiol.00127.2012 22582214 PMC3404833

[69] Keating SE Hackett DA Parker HM O'Connor HT Gerofi JA Sainsbury A. Effect of aerobic exercise training dose on liver fat and visceral adiposity. J Hepatol. (2015) 63:174–82. doi: 10.1016/j.jhep.2015.02.022, PMID: 25863524

[70] Sertie RA Andreotti S Proença AR Campana AB Lima-Salgado TM Batista ML Jr. Cessation of physical exercise changes metabolism and modifies the adipocyte cellularity of the periepididymal white adipose tissue in rats. J Appl Physiol (1985). (2013) 115:394–402. doi: 10.1152/japplphysiol.01272.2012 23703117

[71] Petridou A Tsalouhidou S Tsalis G Schulz T Michna H Mougios V. Long-term exercise increases the DNA binding activity of peroxisome proliferator-activated receptor gamma in rat adipose tissue. Metabolism. (2007) 56:1029–36. doi: 10.1016/j.metabol.2007.03.011, PMID: 17618946

[72] Ricquier D. Respiration uncoupling and metabolism in the control of energy expenditure. Proc Nutr Soc. (2005) 64:47–52. doi: 10.1079/PNS2004408 15877922

[73] Aghapour A Farzanegi P. Effect of six-week aerobic exercise on Chemerin and Resistin concentration in hypertensive postmenopausal women. Electron Physician. (2013) 5:623–30. doi: 10.14661/2013.623-630, PMID: 26120393 PMC4477779

[74] Haus JM Solomon TP Kelly KR Fealy CE Kullman EL Scelsi AR. Improved hepatic lipid composition following short-term exercise in nonalcoholic fatty liver disease. J Clin Endocrinol Metab. (2013) 98:E1181–8. doi: 10.1210/jc.2013-1229, PMID: 23616151 PMC3701282

[75] Nikseresht M Sadeghifard N Agha-Alinejad H Ebrahim K. Inflammatory markers and adipocytokine responses to exercise training and detraining in men who are obese. J Strength Cond Res. (2014) 28:3399–410. doi: 10.1519/JSC.0000000000000553, PMID: 25028994

[76] Hallsworth K Fattakhova G Hollingsworth KG Thoma C Moore S Taylor R. Resistance exercise reduces liver fat and its mediators in non-alcoholic fatty liver disease independent of weight loss. Gut. (2011) 60:1278–83. doi: 10.1136/gut.2011.242073, PMID: 21708823 PMC3152868

[77] Lee S Bacha F Hannon T Kuk JL Boesch C Arslanian S. Effects of aerobic versus resistance exercise without caloric restriction on abdominal fat, intrahepatic lipid, and insulin sensitivity in obese adolescent boys: a randomized, controlled trial. Diabetes. (2012) 61:2787–95. doi: 10.2337/db12-0214, PMID: 22751691 PMC3478522

[78] Bacchi E Negri C Targher G Faccioli N Lanza M Zoppini G. Both resistance training and aerobic training reduce hepatic fat content in type 2 diabetic subjects with nonalcoholic fatty liver disease (the RAED2 randomized trial). Hepatology. (2013) 58:1287–95. doi: 10.1002/hep.26393, PMID: 23504926

[79] Romero-Gómez M Zelber-Sagi S Trenell M. Treatment of NAFLD with diet, physical activity and exercise. J Hepatol. (2017) 67:829–46. doi: 10.1016/j.jhep.2017.05.016 28545937

[80] Zelber-Sagi S Buch A Yeshua H Vaisman N Webb M Harari G. Effect of resistance training on non-alcoholic fatty-liver disease a randomized-clinical trial. World J Gastroenterol. (2014) 20:4382–92. doi: 10.3748/wjg.v20.i15.4382, PMID: 24764677 PMC3989975

[81] Shamsoddini A Sobhani V Ghamar Chehreh ME Alavian SM Zaree A. Effect of aerobic and resistance exercise training on liver enzymes and hepatic fat in Iranian men with nonalcoholic fatty liver disease. Hepat Mon. (2015) 15:e31434. doi: 10.5812/hepatmon.31434, PMID: 26587039 PMC4644631

[82] Slentz CA Bateman LA Willis LH Shields AT Tanner CJ Piner LW. Effects of aerobic vs. resistance training on visceral and liver fat stores, liver enzymes, and insulin resistance by HOMA in overweight adults from STRRIDE AT/RT. Am J Physiol Endocrinol Metab. (2011) 301:E1033–9. doi: 10.1152/ajpendo.00291.2011, PMID: 21846904 PMC3214001

[83] Takahashi A Abe K Usami K Imaizumi H Hayashi M Okai K. Simple resistance exercise helps patients with non-alcoholic fatty liver disease. Int J Sports Med. (2015) 36:848–52. doi: 10.1055/s-0035-1549853, PMID: 26090879

[84] Lillioja S Young AA Culter CL Ivy JL Abbott WG Zawadzki JK. Skeletal muscle capillary density and fiber type are possible determinants of in vivo insulin resistance in man. J Clin Invest. (1987) 80:415–24. doi: 10.1172/JCI113088, PMID: 3301899 PMC442253

[85] Verdijk LB Gleeson BG Jonkers RA Meijer K Savelberg HH Dendale P. Skeletal muscle hypertrophy following resistance training is accompanied by a fiber type-specific increase in satellite cell content in elderly men. J Gerontol A Biol Sci Med Sci. (2009) 64:332–9. doi: 10.1093/gerona/gln050, PMID: 19196907 PMC2655000

[86] Gallagher PM Touchberry CD Teson K McCabe E Tehel M Wacker MJ. Effects of an acute bout of resistance exercise on fiber-type specific to GLUT4 and IGF-1R expression. Appl Physiol Nutr Metab. (2013) 38:581–6. doi: 10.1139/apnm-2012-0301, PMID: 23668768

[87] Oh YS Kim HJ Ryu SJ Cho KA Park YS Park H. Exercise type and muscle fiber specific induction of caveolin-1 expression for insulin sensitivity of skeletal muscle. Exp Mol Med. (2007) 39:395–401. doi: 10.1038/emm.2007.44, PMID: 17603294

[88] Boström P Wu J Jedrychowski MP Korde A Ye L Lo JC. A PGC1-α-dependent myokine that drives brown-fat-like development of white fat and thermogenesis. Nature. (2012) 481:463–8. doi: 10.1038/nature10777 22237023 PMC3522098

[89] Karstoft K Pedersen BK. Skeletal muscle as a gene regulatory endocrine organ. Curr Opin Clin Nutr Metab Care. (2016) 19:270–5. doi: 10.1097/MCO.0000000000000283, PMID: 27101470

[90] Park MJ Kim DI Choi JH Heo YR Park SH. New role of irisin in hepatocytes: the protective effect of hepatic steatosis in vitro. Cell Signal. (2015) 27:1831–9. doi: 10.1016/j.cellsig.2015.04.010, PMID: 25917316

[91] Polyzos SA Kountouras J Anastasilakis AD Geladari EV Mantzoros CS. Irisin in patients with nonalcoholic fatty liver disease. Metabolism. (2014) 63:207–17. doi: 10.1016/j.metabol.2013.09.013 24140091

[92] Kim HJ Lee HJ So B Son JS Yoon D Song W. Effect of aerobic training and resistance training on circulating irisin level and their association with change of body composition in overweight/obese adults: a pilot study. Physiol Res. (2016) 65:271–9. doi: 10.33549/physiolres.932997, PMID: 26447516

[93] LeBrasseur NK Kelly M Tsao TS Farmer SR Saha AK Ruderman NB. Thiazolidinediones can rapidly activate AMP-activated protein kinase in mammalian tissues. Am J Physiol Endocrinol Metab. (2006) 291:E175–81. doi: 10.1152/ajpendo.00453.2005, PMID: 16464908

[94] Mul JD Stanford KI Hirshman MF Goodyear LJ. Exercise and regulation of carbohydrate metabolism. Prog Mol Biol Transl Sci. (2015) 135:17–37. doi: 10.1016/bs.pmbts.2015.07.020, PMID: 26477909 PMC4727532

[95] Tanase DM Gosav EM Costea CF Ciocoiu M Lacatusu CM Maranduca MA. The intricate relationship between type 2 diabetes mellitus (T2DM), insulin resistance (IR), and nonalcoholic fatty liver disease (NAFLD). J Diabetes Res. (2020) 2020:1–16. doi: 10.1155/2020/3920196 PMC7424491 32832560

[96] Anania C Perla FM Olivero F Pacifico L Chiesa C. Mediterranean diet and nonalcoholic fatty liver disease. World J Gastroenterol. (2018) 24:2083–94. doi: 10.3748/wjg.v24.i19.2083, PMID: 29785077 PMC5960814

[97] Plaz Torres MC Aghemo A Lleo A Bodini G Furnari M Marabotto E. Mediterranean diet and NAFLD: what we know and questions that still need to be answered. Nutrients. (2019) 11:2971. doi: 10.3390/nu11122971, PMID: 31817398 PMC6949938

[98] Sekiya M Yahagi N Matsuzaka T Najima Y Nakakuki M Nagai R. Polyunsaturated fatty acids ameliorate hepatic steatosis in obese mice by SREBP-1 suppression. Hepatology. (2003) 38:1529–39. doi: 10.1016/j.hep.2003.09.028, PMID: 14647064

[99] Mirabelli M Chiefari E Arcidiacono B Corigliano DM Brunetti FS Maggisano V. Mediterranean diet nutrients to turn the tide against insulin resistance and related diseases. Nutrients. (2020) 12:1066. doi: 10.3390/nu12041066, PMID: 32290535 PMC7230471

[100] Papakonstantinou E Oikonomou C Nychas G Dimitriadis GD. Effects of diet, lifestyle, Chrononutrition and alternative dietary interventions on postprandial Glycemia and insulin resistance. Nutrients. (2022) 14:823. doi: 10.3390/nu14040823, PMID: 35215472 PMC8878449

[101] MASLD. EASL-EASD-EASO clinical practice guidelines on the management of metabolic dysfunction-associated steatotic liver disease (MASLD). J Hepatol. (2024). doi: 10.1016/j.jhep.2024.04.031 38851997

[102] Ryan MC Itsiopoulos C Thodis T Ward G Trost N Hofferberth S. The Mediterranean diet improves hepatic steatosis and insulin sensitivity in individuals with non-alcoholic fatty liver disease. J Hepatol. (2013) 59:138–43. doi: 10.1016/j.jhep.2013.02.012, PMID: 23485520

[103] Hardie DG. AMP-activated protein kinase: maintaining energy homeostasis at the cellular and whole-body levels. Annu Rev Nutr. (2014) 34:31–55. doi: 10.1146/annurev-nutr-071812-161148, PMID: 24850385 PMC5693323

[104] Cantó C Gerhart-Hines Z Feige JN Lagouge M Noriega L Milne JC. AMPK regulates energy expenditure by modulating NAD+ metabolism and SIRT1 activity. Nature. (2009) 458:1056–60. doi: 10.1038/nature07813, PMID: 19262508 PMC3616311

[105] van Loon LJ Greenhaff PL Constantin-Teodosiu D Saris WH Wagenmakers AJ. The effects of increasing exercise intensity on muscle fuel utilisation in humans. J Physiol. (2001) 536:295–304. doi: 10.1111/j.1469-7793.2001.00295.x, PMID: 11579177 PMC2278845

[106] Wasserman DH Zinman B. Exercise in individuals with IDDM. Diabetes Care. (1994) 17:924–37. 7956645 10.2337/diacare.17.8.924

[107] Romijn JA Coyle EF Sidossis LS Gastaldelli A Horowitz JF Endert E. Regulation of endogenous fat and carbohydrate metabolism in relation to exercise intensity and duration. Am J Phys. (1993) 265:E380–91. doi: 10.1152/ajpendo.1993.265.3.E380, PMID: 8214047

[108] Arner E Westermark PO Spalding KL Britton T Rydén M Frisén J. Adipocyte turnover: relevance to human adipose tissue morphology. Diabetes. (2010) 59:105–9. doi: 10.2337/db09-0942, PMID: 19846802 PMC2797910

[109] Arner P Kriegholm E Engfeldt P Bolinder J. Adrenergic regulation of lipolysis in situ at rest and during exercise. J Clin Invest. (1990) 85:893–8. doi: 10.1172/JCI114516, PMID: 2312732 PMC296507

[110] Dubé JJ Amati F Stefanovic-Racic M Toledo FG Sauers SE Goodpaster BH. Exercise-induced alterations in intramyocellular lipids and insulin resistance: the athlete's paradox revisited. Am J Physiol Endocrinol Metab. (2008) 294:E882–8. doi: 10.1152/ajpendo.00769.2007, PMID: 18319352 PMC3804891

[111] van Loon LJ Jeukendrup AE Saris WH Wagenmakers AJ. Effect of training status on fuel selection during submaximal exercise with glucose ingestion. J Appl Physiol (1985). (1999) 87:1413–20. doi: 10.1152/jappl.1999.87.4.1413 10517772

[112] Bruce CR Hoy AJ Turner N Watt MJ Allen TL Carpenter K. Overexpression of carnitine palmitoyltransferase-1 in skeletal muscle is sufficient to enhance fatty acid oxidation and improve high-fat diet-induced insulin resistance. Diabetes. (2009) 58:550–8. doi: 10.2337/db08-1078, PMID: 19073774 PMC2646053

[113] Koves TR Ussher JR Noland RC Slentz D Mosedale M Ilkayeva O. Mitochondrial overload and incomplete fatty acid oxidation contribute to skeletal muscle insulin resistance. Cell Metab. (2008) 7:45–56. doi: 10.1016/j.cmet.2007.10.013 18177724

